# Ischemic modified albumin as a new biomarker in predicting oxidative stress in alopecia areata

**DOI:** 10.3906/sag-1708-35

**Published:** 2019-02-11

**Authors:** Hatice ATAŞ, Müzeyyen GÖNÜL, Yasin ÖZTÜRK, Mustafa KAVUTÇU

**Affiliations:** 1 Department of Dermatology, Dışkapı Yıldırım Beyazıt Training and Research Hospital, University of Health Sciences, Ankara Turkey; 2 Department of Biochemistry, Faculty of Science, Gazi University, Ankara Turkey

**Keywords:** Biomarkers, alopecia areata, oxidative stress, antioxidative system, ischemia-modified albumin

## Abstract

**Background/aim:**

Results show that oxidative stress is a pathophysiologic factor for alopecia areata (AA); however, the markers used can be confounding. Thus, we aimed to investigate the role of oxidative stress in the pathogenesis of AA through an evaluation of ischemia-modified albumin (IMA); other markers of the oxidant/antioxidant system, such as SOD, CAT, GSH-ST, and MDA; and contributing clinical risk factors.

**Materials and methods:**

The usefulness of IMA as a new marker for oxidative stress was compared with that of other markers and evaluated in patients with AA.

**Results:**

The mean serum level of IMA was of higher statistical significance in AA patients than in the control group (IMA: 0.57 ± 0.01 vs. 0.52 ± 0.02 ΔABSU, P < 0.0001). IMA (P = 0.03, OR = 25.8, 95% CI = 1.4–482.7) was found to be an independent predictor of oxidative stress in patients with AA. Increased severity of AA was found as an independent risk factor for IMA.

**Conclusion:**

Long-lasting disease, male sex, >1 site of involvement of disease, and increased severity of disease were correlated with increased oxidation. Presence of AA, male sex, and severe disease were determined to be independent risk factors for antioxidant and oxidant systems. IMA has great potential as a biomarker of oxidative stress in AA when compared to other studied biomarkers.

## 1. Introduction

The skin is an organ that protects against environmental changes. Skin symptoms may occur concurrent with or prior to major systemic internal diseases such as systemic lupus erythematosus, dermatomyositis, Sjögren syndrome, hyperthyroidism, acrodermatitis enteropathica, and scurvy. Alopecia areata (AA) is one of the cutaneous manifestations of these diseases (1,2).

Characterized by loss of hair on the scalp or any other part of the body, AA is a skin disease seen in 1.7% of the population during their lifetimes (3). Certain pathogenesis and etiologies of AA are unknown, but autoimmunity, emotional stress, increased levels of inflammatory cytokines, oxidative stress, and activation of the hypothalamic–pituitary–adrenal axis are considered associated factors in etiology (2,4,5). Comprehensive studies evaluating oxidant/antioxidant status in AA have been reported (6,7). The results show that oxidative stress is a pathophysiologic factor for AA; however, the markers used can be confounding. Therefore, there remains a requirement for novel, highly sensitive, and specific biomarkers.

Ischemia-modified albumin (IMA), calculated through the evaluation of the binding of cobalt to albumin, is a new biomarker for ischemia. A level of IMA is also detected in diseases related to oxidative stress, such as psoriasis, vascular injury of diabetes mellitus, multiple sclerosis, some cancers, acute appendicitis, polycystic ovary syndrome, and beta-thalassemia major (8–14). However, IMA has not yet been evaluated in AA. Accordingly, we aimed to evaluate the serum IMA level, compared with other markers, in patients with AA as a useful marker for assessing oxidative stress in this disease.

## 2. Materials and methods

### 2.1. Subjects

This is a prospective single-center, matched case-control study. A total of 120 subjects were separated into two groups: 1. Patient group = subjects with AA; 2. Control group = subjects without AA. We selected 60 patients with AA (30 females and 30 males with a mean age of 28.1 ± 5.3 [19–39] years), and 60 age- and sex-matched controls (30 females and 30 males with a mean age of 28.2 ± 5.8 [19–40] years) (Table 1). 

**Table 1:** Comparison of clinical and laboratory features between controls and patients.

			Patients (n = 60)			Controls (n=60)			p
Sex	Male Female		30 (50%) 30 (50%)			30 (50%) 30 (50%)			1.00
Localization	Scalp Beard Scalp-beard-nails Scalp-beard Scalp-nails		30 (50%) 12 (20%) 9 (15%) 5 (8.4%) 4 (6.6%)						
Involvement	≤1 site≥2 sites		42 (70%) 18 (30%)						
Severity of disease	Mild Moderate Severe		26(46.1%) 24 (38.5%) 10 (15.4%)						
Classification of disease S††B*N**	S0B1N0 S1B0N0 S1B0N1 S2B0N0 S2B0N1 S2B1N0 S2B1N1 S5B0N0 B5B2N1		12 (19.2%) 23 (38.5%) 2 (3.8%) 5 (7.7%) 2 (3.8%) 5 (7.7%) 2 (3.8%) 2 (3.8%) 7 (11.5%)						
		Mean	±SD‡‡	CV	Range	Mean	CV	Range	P
Age	years	28.1	5.3		19–39	28.2	5.8	19–40	0.90
Duration of disease	months	4.5	1.9		1–8				
SOD (IU/mL) §§	Total	1.6	±0.2	0.13	1.2–1.9	1.8	0.33	0.7–2.8	0.035
CAT (IU/mL) †	Total	20.4	±4.8	0.24	12.8–33.2	25.1	0.23	12.8–38.3	0.005
GSH-ST (IU/mL) ‡	Total	3.1	±0.4	0.13	2.1–3.9	2.5	0.24	1.4–3.9	0.002
MDA (nmol/L) ||	Total	35.1	±5.6	0.16	26.4–45.1	23.6	0.17	17.2–32.3	<0.0001
IMA (ΔABSU) §	Total	0.57	±0.01	0.2	0.56–0.6	0.52	0.38	0.5–0.57	<0.0001

* Body, †catalase, ‡glutathione-S-transferase, § ischemia modified albumin, || malondialdehyde, **nails, ††scalp, ‡‡ standard deviation, §§superoxide dismutase. Coefficient of variation (CV) was defined as the ratio of the SD to the mean.

The subjects were admitted to the outpatient clinic of dermatology for medical examination. Alopecia areata was diagnosed based on clinical and, if necessary, histopathological findings. Background pathophysiological data, lifestyles, and lifestyle-related diseases were investigated. Inclusion criteria for patients were as follows: a) presence of AA, b) age 18 years or older, c) newly diagnosed patient. Selected cases were newly diagnosed. Cases under treatment were excluded. Controls over 18 years of age were recruited from among patients who had been referred for minor dermatological problems, such as nevus, to avoid biasing the study results. 

Immunocompromised patients and subjects with autoimmune disorders, diabetes mellitus, familial hypercholesterolemia, neoplastic diseases, obesity, liver and kidney diseases, and recent major surgical procedures were excluded. Subjects taking diuretics or hormone replacement therapy, those who used alcohol or smoked, those who practiced excessive exercise apart from daily life activities, and those taking any treatment, including vitamins and antiinflammatory drugs, in the last 3 months were also excluded. Approval for the study was obtained from the institutional ethics committee. Informed written consent was obtained from all subjects.

### 2.2. Disease severity

Disease severity was graded as mild, moderate, or severe (15).

·Mild: ≤3 patches with diameter of ≤3 cm or disease limited to eyelashes and eyebrows.

·Moderate: >3 patches with diameter of >3 cm without alopecia totalis or universalis.

·Severe: The presence of alopecia totalis or universalis.

### 2.3. Localization and classification of AA

Alopecia areata was classified according to localization as follows: 

·Scalp: S0 = no hair loss, S1 = <25% hair loss, S2 = 25%–49% hair loss, S3 = 50%–74% hair loss, S4 = 75%–99% hair loss (a = 75%–95%, b = 96%–99%), S5 = 100% hair loss.

·Body hair: B0 = none, B1 = some, B2 = 100% hair loss.

·Nails: N0: none, N1 = some, N1a = 20-nail dystrophy (e.g., S4bB1N0 = 96% loss of scalp hair with beard, but no involvement of other body hair or nails) (16).

### 2.4. Chemicals and enzyme analyses

Samples were obtained from participants after overnight fasting of at least 12 h. After 30 min, they were centrifuged at 4 °C (1000 rpm, 15 min) before being stored at –80 °C for no longer than 3 months. The samples were blood, but the frozen product was serum. In this condition and time frame, no meaningful differences were detected between parameters. 

Superoxide dismutase (SOD), catalase (CAT), and glutathione-S-transferase (GSH-ST) activities and levels of malondialdehyde (MDA) and IMA were tested in both patients and controls. All procedures were performed at 4 °C throughout the experiment. All enzyme, MDA, and IMA procedures were performed manually without using commercial kits. 

SOD, CAT, and GSH-ST enzyme analyses were performed as described by Durak, Aebi, and Habig, respectively (17–19). 

The assay mixture for the SOD activity method contained 0.30 mM xanthine, 0.60 mM EDTA, 150 µM nitro blue tetrazolium (NBT), 400 mM Na2CO3, 167 U/L xanthine oxidase, 1.0 g/L BSA, 8 mM CuCl2, 150 mM NaCN, and 100 µL of the sample added to 1.0 mL of the assay mixture and vortexed. The SOD activity method was based on the measurement of absorbance increase at 560 nm, due to the reduction of NBT to NBTH2 at the end of 20 min. One unit of SOD activity was defined as the enzyme protein amount that caused 50% inhibition in the NBTH2 reduction rate. 

The CAT activity method was based on the measurement of the absorbance decrease due to H2O2 consumption at 240 nm. In the ultraviolet range, H2O2 shows a continual increase in absorption with a decreasing wavelength. The decomposition of H2O2 can be followed directly by the decrease in the absorbance at 240 nm. The difference in absorbance (∆A 240) per unit time is a measure of CAT activity. The reagents used were a phosphate buffer (50 mmol/L, pH 7.0) and 30 mmol/L H2O2 in a phosphate buffer, which was prepared fresh before each assay. The reaction was initiated by the addition of 1 mL of 30 mmol/L H2O2 to 20 µL of sample. A blank assay with buffer instead of substrate and 20 µL of sample were used to correct for any nonenzymatic reaction. The absorbance was observed for approximately 180 s. 

Finally, the GSH-ST activity method was based on the measurement of absorbance changes at 340 nm due to the formation of a GSH-CDNB complex. One unit of GSH-ST activity is defined as the amount of enzyme producing 1 mmol of GSH-CDNB conjugate/min under the conditions of the assay.

The MDA assay was carried out using the thiobarbituric acid (TBA) method to determine lipid peroxidation (20). Thiobarbituric acid reactive substances (TBARS) measurements were conducted based on the reaction of MDA with TBA, which forms a pink pigment with an absorption maximum at 532 nm in acid pH, while 1,1,3,3-tetraethoxypropane was used as a standard MDA solution. 

The IMA assay was determined by a manual colorimetric assay described by Bar-Or et al. (21), the Co(II)-albumin binding assay. This method consists of adding a known amount of exogenous Co(II) to a plasma sample and measuring unbound Co(II) spectrophotometrically using dithiothreitol (DTT). 

Enzyme activities and MDA and IMA levels were determined by continuously monitoring end-point changes in absorbance at 25 °C using a Shimadzu UV-1601 spectrophotometer (Kyoto, Japan); the results were expressed in IU/mL for CAT and GSH-ST enzymes, while SOD activities were given in U/mL. The MDA and IMA results were given as nmol and mL, respectively, and absorbance difference units were denoted between the blank and the sample (ΔABSU).

### 2.5. Data analysis

SPSS 15.0 was used for statistical analysis; mean ± standard deviation (SD), median, coefficient of variation (CV), and range of frequencies were calculated for the variables. The variables were investigated using an analytical method (Kolmogorov–Smirnov test) to determine whether they were normally distributed. Chi-square tests or Fisher’s exact tests (when chi-square test assumptions did not hold due to low expected cell counts) were used where appropriate to compare proportions of the sexes between patients and controls. Mann–Whitney U tests (not normally distributed) or Student’s t-tests (normally distributed) were used where appropriate to compare age, SOD, CAT, GSH-ST, MDA, and IMA between patients and controls. Pearson or Spearman tests were used for correlation analysis. For the multivariate analysis, possible factors such as SOD, CAT, GSH-ST, MDA, and IMA and covariates such as sex and age identified with univariate analyses were further entered into the logistic regression analysis to determine independent predictors of AA. 

We next considered which independent predictors were effective on levels of SOD, CAT, GSH-ST, MDA, and IMA. For this, multivariate linear regression models were evaluated for various factors such as age, sex, duration, involvement, localization, classification, and severity of disease as independent predictors of antioxidant and oxidant systems. The capacity of the serum’s IMA value for predicting the presence of oxidative stress was analyzed using ROC (receiver operating characteristics) curve analysis. When a significant cut-off value was observed, the sensitivity, specificity, and positive and negative predictive values were presented. When evaluating the area under the curve, a 5% type-I error was used to accept a statistically significant predictive value of the test variables. 

## 3. Results

### 3.1. Demographic results

Certain characteristics and biochemical results of the subjects are shown in Tables 1 and 2. The mean duration of the disease was 4.5 ± 1.9 (1–8) months. Involvement of AA was detected only in the scalp in 30 (50%) patients; only in the beard in 12 (20%) patients; in the scalp, beard, and nails in 9 (15%) patients; in the scalp and beard in 5 (8.4%) patients; and in the scalp and nails in 4 (6.6%) patients. One site of localization was found in 42 (70%) patients. Severity of AA was classified as mild in 26 (46.1%) patients, moderate in 24 (38.5%) patients, and severe in 10 (15.4%) patients. Classification of AA was evaluated as S0B1N0 in 12 (19.2%), S1B0N0 in 23 (38.5%), S1B0N1 in 2 (3.8%), S2B0N0 in 5 (7.7%), S2B0N1 in 2 (3.8%), S2B1N0 in 5 (7.7%), S2B1N1 in 2 (3.8%), S5B0N0 in 2 (3.8%), and B5B2N1 in 7 (11.5%) patients. 

**Table 2:** Comparison of antioxidant enzymes and oxidant biomarkers according to disease severity.

	Severity	Mean	±SD	Range	P*
SOD (IU/ml) **	Mild	1.5	0.27	1–1.9	0.28
	Moderate	1.5	0.11	1.3–1.7	
	Severe	1.4	0.16	1.2–1.6	
CAT (IU/ml) †	Mild	22.8	8.8	12.8–43.4	0.86
	Moderate	24.3	7.1	15.3–34.3	
	Severe	25.2	7.7	17.9–33.2	
GSH-ST (IU/ml) ‡	Mild	3.0	0.36	2.6–3.8	0.62
	Moderate	3.1	0.5	2.1–3.9	
	Severe	2.8	0.4	2.5–3.5	
MDA (nmol/L) ||	Mild	30.4	5.1	22.4–42.6	0.30
	Moderate	33.0	8.8	24.3–42.4	
	Severe	35.3	7.1	23.8–45.1	
IMA (ΔABSU) §	Mild	0.56	0.007	0.55–0.58	0.004
	Moderate	0.57	0.008	0.56–0.60	
	Severe	0.58	0.012	0.57–0.59	

* P < 0.05 is significant, † catalase, ‡ glutathione-S-transferase, § ischemia-modified albumin, || malondialdehyde, ** superoxide dismutase.

### 3.2. Biochemical results

Mean serum enzyme activity of SOD and CAT, excluding GSH-ST, was lower in the patient group than in the control group. Serum levels of MDA and IMA were higher in the patient group than in the control group (SOD: 1.6 ± 0.2 vs. 1.8 ± 0.6 IU/mL, P = 0.035; CAT: 20.4 ± 4.8 vs. 25.1 ± 5.9 IU/mL, P = 0.005; GSH-ST: 3.1 ± 0.4 vs. 2.5 ± 0.6 IU/mL, P = 0.002; MDA: 35.1 ± 5.6 vs. 23.6 ± 4.1 nmol/L, P < 0.0001; IMA: 0.57 ± 0.01 vs. 0.52 ± 0.02 ΔABSU, P < 0.0001). 

### 3.3. Correlations

Some correlations were detected between duration of disease and IMA (r = 0.43, P = 0.03), MDA and IMA (r = 0.64, P < 0.0001), sex and CAT (r = 0.56, P = 0.003), sex and MDA (r = –0.54, P = 0.004), localization and disease severity (r = 0.58, P = 0.002), localization and MDA (r = 0.48, P = 0.014), and disease severity and IMA (r = 0.63, P = 0.001). 

### 3.4. Predictors and risk factors

Levels of SOD (P = 0.04, OR = 0.003, 95% CI = 0.0001–0.9) and IMA (P = 0.03, OR = 25.8, 95% CI = 1.4–482.7) were found to be independent predictors of oxidative stress in patients with AA in the multivariate analysis (see Table 3). IMA was found to be significant according to disease severity (P = 0.004). Presence of AA and sex for SOD, sex for CAT and MDA, presence of AA for GSH-ST, and duration and severity of disease for IMA were found to be independent risk factors in a multivariate analysis (see Table 4). 

**Table 3:** Effects of SOD, CAT, GST, MDA, IMA, age, and sex on oxidative stress in patients with vitiligo in the multivariate analysis.

			Univariate analysis			Multivariate analysis		
Predictor		Category	P*	OR§§	95% CI ‡‡	P	OR	95% CI
SOD††	U/mL		0.03	0.24	0.07–0.87	0.04	0.003	0.0001–0.9
CAT†	U/mL		0.48	0.97	0.9–1.1			
GSH-ST§	U/mL		0.004	5.4	1.7–17.5			
MDA**	nmol/L		<0.0001	1.3	1.1–1.6			
IMA||	ΔABSU		<0.0001	5.4	2.1–13.5	0.03	25.8	1.4–482.7
Age	Years		0.90	0.9	0.9–1.1			
Sex		M/F	0.90	1.1	0.38–3.1			

* P < 0.05: significant, †catalase, ‡ confidence interval, § glutathione-S-transferase, || ischaemia-modified albumin; ** malondialdehyde, †† superoxide dismutase. ‡‡Confidence intervals were estimated using the logistic regression model. §§ Odds ratio was estimated using the logistic regression model. Simple first was selected to evaluate the effect of the categorical predictors.

**Table 4:** Independent effect of different predictors on antioxidant enzyme and oxidant biomarkers.

Dependent variables	β 0‡‡	Independent variables	β 1§§	p	95% CI‡ for β
Antioxidant system					
SOD (IU/mL) ††	1.2	Presence of AA*	–0.23	0.04	–0.5–(–0.007)
		Female sex	0.39	0.001	0.16–0.62
CAT (IU/mL) †	9.4	Female sex	10.6	<0.0001	5.5–15.7
GSH-ST (IU/mL) §	2.5	Presence of AA	0.49	0.001	0.2–0.8
Oxidant system					
MDA (nmol/L) **	42.6	Female sex	–7.4	0.004	–12.2–(–2.5)
IMA (ΔABSU) ||	0.55	Severity of disease	0.008	0.004	0.003–0.013

*Alopecia areata, † catalase, ‡ confidence interval, § glutathione-S-transferase, || ischemiamodified albumin, **malondialdehyde, †† superoxide dismutase, ‡‡ constant, §§ regression coefficient.

In female subjects, enzyme activities of SOD and CAT were increased while MDA levels were decreased (SOD: 1.9 ± 0.5 vs. 1.5 ± 0.4 IU/mL, P = 0.001; CAT: 26.6 ± 6.9 vs. 22.7 ± 6.1 IU/mL, P = 0.027; MDA: 25.2 ± 4.8 vs. 29.7 ± 7.8 nmol/L, P = 0.011). 

### 3.5. Predictivity 

IMA was found to be of significant value in predicting the presence of oxidative stress in patients with AA (AUC: 0.97, 95% CI: 0.93–0.99, P < 0.0001) and was higher than the other studied biomarkers. The sensitivity, specificity, and positive and negative predictive values of IMA were 96%, 94%, 93%, and 97% for 0.55 ΔABSU, respectively. These were also higher than the other studied biomarkers (Table 5; Figure).

**Table 5:** Capacity of biomarkers in the evaluation of oxidative stress in alopecia areata.

		AUC*	P	95% CI‡	Cut-off	Sensitivity	Specificity	Positive predictive value	Negative predictive value
						%	%	%	%
SOD††	U/mL	0.68	0.018	0.53–0.84	1.83	96	62	67	95
CAT†	IU/mL	0.58	0.27	0.43–0.74	22.8	65	65	61	69
GSH-ST§	IU/mL	0.74	0.002	0.61–0.87	2.8	73	71	68	76
MDA**	nmol/mL	0.88	<0.0001	0.80–0.97	26.1	85	77	76	86
IMA||	ΔABSU	0.97	<0.0001	0.93–0.99	0.55	96	94	93	97

* Area under the curve, † catalase, ‡ confidence interval, § glutathione-S-transferase, || ischaemia-modified albumin, **malondialdehyde, †† superoxide dismutase.

**Figure 1:**
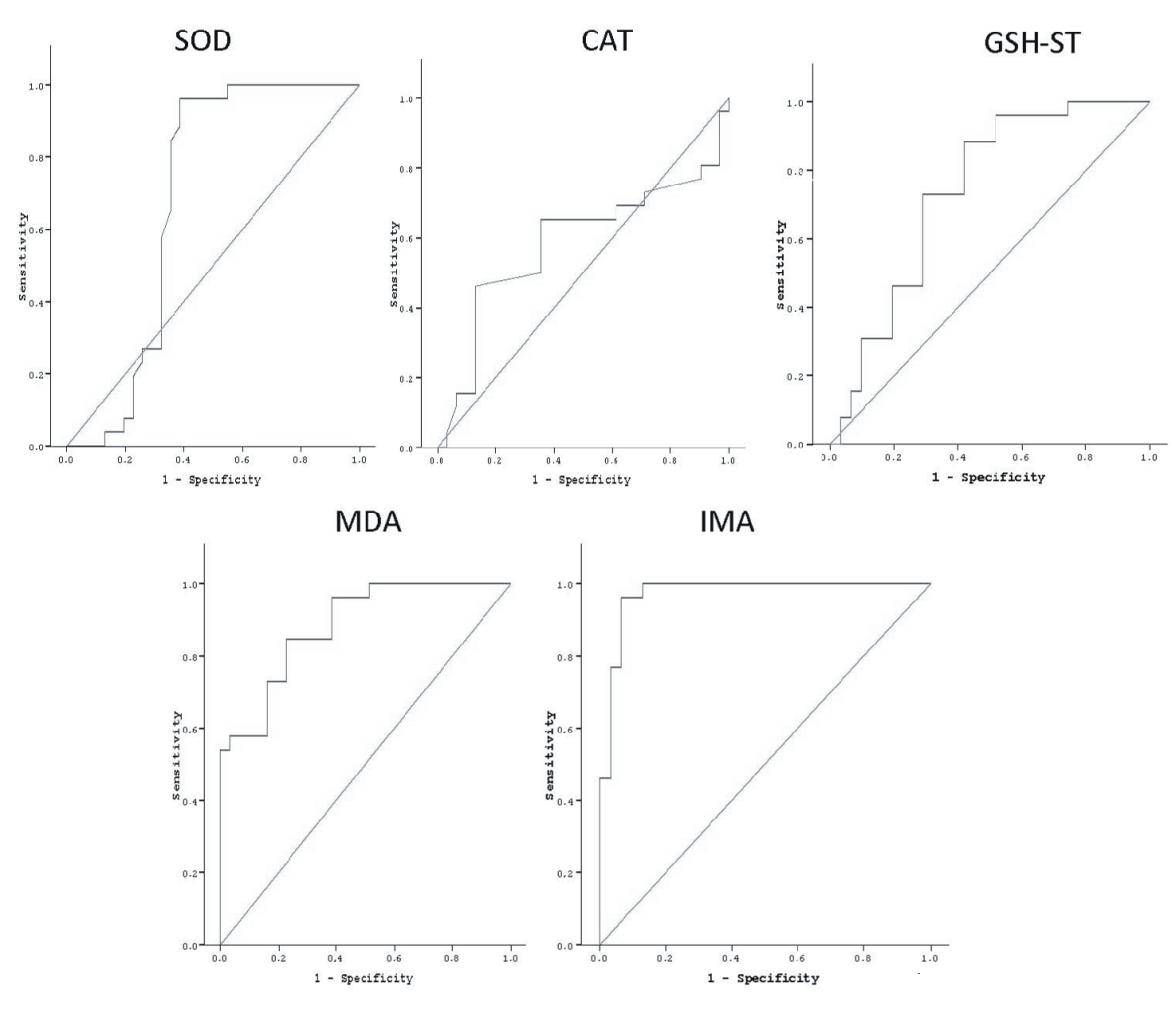
Serum SOD, CAT, GSH-ST, MDA, and IMA values were recorded in patients with alopecia areata (AA) and controls. It was investigated whether SOD, CAT, GSH-ST, MDA, and IMA were diagnostic and predictive for oxidative stress in AA. Cut-off values were evaluated with receiver operating characteristic (ROC) curve. After ROC analysis, serum IMA was found the most diagnostic marker predicting oxidative stress in patients with AA (AUC: 0.97, 95% CI: 0.93–0.99, P < 0.0001).

## 4. Discussion

Oxidative stress is one main theories for the pathogenesis of AA (6,7). As in many diseases, the prognostic role of biomarkers has been widely investigated. The approach used with the patient can be dictated by the cut-off level of biomarkers from evaluation to treatment; therefore, high sensitivity and specificity of biomarkers are necessary features for more timely prediction of the disease. This research clearly demonstrates that increases in serum IMA are closely related to oxidative stress in AA. An increase in said stress may be a key contributor to the pathogenesis and progression of the condition.

Autoimmunity, emotional stress, increased levels of inflammatory cytokines, oxidative stress, and activation of the hypothalamic–pituitary–adrenal axis are considered associated factors in etiologies of AA (2,4,5). While exogenous stimuli (such as UV irradiation, trauma, and stress) and endogenous stimuli (such as cellular metabolism, proliferation, differentiation, apoptosis, and immune reactions) enhance reactive oxygen species (ROS) accumulation, enzymatic antioxidants (such as CAT, SOD, and GSH-ST), nonenzymatic antioxidants (such as β-carotene, ubiquinone, vitamin E, and vitamin C), and antioxidant pathways (such as Nrf2-ARE/HO-1 and Forkhead box class O [FOXO]) inhibit the production of ROS (22–24). Some differential features, such as relatively asymptomatic, less inflammatory nonheterologous infiltrate, contain T cells and include deep dermis involvement and impaired antioxidant defenses in AA compared to other inflammatory diseases (6,25). Patients with AA have an impaired antioxidant defense, while active shedding due to aging or androgens is related to a decrease in activity of antioxidant defense and an increase in ROS (26). In summary, impaired antioxidant defenses have certain effects, such as reduction of ROS in AA.

Although some studies have investigated antioxidative enzymes and oxidative bioproducts in AA, their results are controversial (6,7). Comparative studies of oxidative stress in AA that evaluate both antioxidative and oxidative systems are very rare. Some studies reported dysfunction of antioxidant systems such as SOD and CAT enzymes (27–31). In addition, increased total oxidant capacity (TOC), oxidative stress index (OSI), and decreased total antioxidant capacity (TAC) were documented by Bakry et al. (6). Decreased CAT activity due to CAT gene mutation with H2O2 accumulation has also been reported (32). Mitochondrial alterations with oxidative stress can lead to the opening of mitochondrial pores, alteration of cellular redox states, apoptosis, decrease in CAT activity, and increase in SOD activity as a compensatory mechanism (33–35).

Our study showed significant changes in the blood of AA patients. In our study, activities of CAT and SOD were lower than in controls. These results showed that insufficient response to oxidative stress was a major cause of AA. A decrease in enzymatic SOD and CAT activities may be associated with direct oxidative damage and changes in gene expression. They fight against free radicals first. In our study, SOD was found to be protective (P = 0.04, OR = 0.003, 95% CI: 0.0001–0.9). SOD enzyme activity depends on cofactors such as zinc, manganese, and copper; however, we did not evaluate these in our study. Decreased SOD enzyme activity may cause increased levels of superoxide radicals. Thus, oxidative stress and its harmful effect may worsen. The body could not manage to compensate oxidative stress with the antioxidant system, as the only source of H2O2 in the organism may not be due to a reaction catalyzed by SOD. Despite the decrease in SOD and CAT activities, H2O2 can be produced by alternative pathways (xanthine oxidase and urate oxidase) or by a decrease in GSH-Px enzyme activity. 

Polymorphism of the GSH-ST gene reduces its activity (32,36,37). This gene catalyzes the binding of glutathione (GSH) to free radicals. It is inactive for H2O2 and reduces organic hydroperoxide in the body if necessary. Increased activity of GSH-ST, with the aim of catalyzation without polymorphism, can contribute to normal increases in nucleophilic effects of GSH. Thus, the merging of oxidative bioproducts with other macromolecules in the body and their harmful effects can be prevented. Increased enzyme activity of GSH-ST was found in our study. The level of GSH could help us, but we did not evaluate it. Increased GSH-ST can reduce the ROS with GSH. This is a reaction to compensate for ROS, and thus these factors may explain the increased enzyme activity of GSH-ST against oxidation. 

The level of MDA, which indicates oxidative stress, was higher in AA patients than in controls (27,29). Severity and longer duration of the disease can increase the level of MDA (27,38). It is correlated with increased oxidative stress of tissue. In our study, increased oxidative bioproducts such as IMA and MDA due to ROS were detected. Dysfunction of the antioxidant system in AA can explain these mechanisms. An increase in the amount of MDA, which is a lipid peroxidation product, supports this notion. In our study, however, the capacity of MDA was lower than that of IMA when it came to predicting oxidative stress in AA. In addition, IMA was found to be a significant biomarker according to the severity of the disease (P = 0.004).

Albumin binds cations or anions weakly and reversibly, despite negatively charged protein. It controls the levels of heavy metals in the blood. IMA measured by the albumin cobalt binding test is a new biomarker for ischemia. In ischemic status, albumin loses binding capacity and ability of transition metals on N-terminal ends due to aspartic acid deletion (21). Although an increased level of IMA is also detected in diseases related to oxidative stress, the level of IMA in AA patients has not yet been investigated. In our study, an increased amount of IMA reinforced the oxidative stress in AA. It is important to determine which patients are at high risk at the time of admission. According to our study, the sensitivity, specificity, and positive and negative predictive values and capacity of IMA were 96%, 94%, 93%, and 97% for 0.55 ΔABSU, respectively. These were higher than the values of SOD, CAT, GSH-ST, and MDA. A level of IMA above 0.55 ΔABSU may be evaluated as indicating high risk of oxidative stress in AA. In our study, IMA had a close association with oxidative stress in AA (P = 0.03, OR = 25.8, 95% CI = 1.4–482.7). Antioxidative prophylaxis can be used for these patients.

Oxidative stress has some adverse impacts on organs and tissues beyond the skin, including cardiovascular diseases, diabetes, aging in humans, subfertility, psychiatric diseases, Alzheimer disease, Parkinson disease, progression of cancer, and epilepsy (39–42). Although significant changes were observed in the blood of AA patients, we did not investigate adverse impacts of oxidative stress. These diseases can be evaluated in AA. In certain circumstances, the use of antioxidant prophylaxis may be effective for preventing the progression of diseases (43).

In the cases of disease, remission is important in controlling balance in both the immune system and the oxidative/antioxidative system. Proper processing of the antioxidant system and support will contribute to the correction of impaired metabolic balance and inhibit the generation of inflammatory mediators, cytokines, and growth factors due to oxidative stress (44). It is difficult to prescribe certain drugs that offer protection from oxidant products, as there must be agreement from the bioethical commission. However, initiatives to strengthen the antioxidant system of AA patients can provide significant contributions to courses of treatment. Vitamins A, E, and C, within normal limits, may be supplied for use without hazardous side effects. To investigate this further, it is necessary to carry out studies in conjunction with antioxidant therapy.

In positive correlations between oxidative stress biomarkers (IMA and MDA) and severity and longer duration of disease, involvement was found above one site of localization. In addition, there was a positive correlation between MDA and IMA. Dissemination of disease, disease lasting a long period of time, and severe disease were associated with a high level of oxidative stress. Increased CAT activity and a decreased level of MDA correlated with female sex. Some interpretations should be given regarding the importance of sex. Juvenile AA patients had more stressful life events, higher NK cell levels and 24-h urine catecholamines, and lower IgA and 24-h urine free cortisol (45). Higher baseline and lower reactive sympathetic activity as well as chronicity—rather than acuteness—of the condition can explain this result. Low-level cortisol may be associated less with disturbance of the hypothalamic–pituitary–adrenal axis in AA than with childhood trauma. IgA levels are more sensitive than cortisol levels, and the protective effect of IgA is decreased in AA. Single-parent families and less expressiveness are other features of juvenile AA patients (45). Murali and Chen (46) reported that males had reduced cortisol reactivity compared with females. In this study, enzyme activity of females was higher than that of males. Thus, a decreased level of MDA is normal in females. Neuroendocrine and immunological parameters also affect this result. The antioxidant system tries to set body homeostasis to a greater degree in females than in males.

The presence of AA, female sex, and severity of disease were found to be effective independent factors when we analyzed antioxidant and oxidant systems. In females, increased enzyme activity such as CAT contributed to a decreased level of MDA. Presence of AA decreased SOD activity due to direct oxidative damage and changes in gene expression. Dysfunction of enzyme activity can be effective on this reduction. GSH-ST activity was increased by presence of AA. Increased GSH-ST can try to reduce the ROS due to continuing oxidative stress with GSH. This is a reaction to compensate for the ROS. These factors may explain the increased enzyme activity of GSH-ST against oxidation in the presence of AA. Increased oxidative stress due to severity of disease increased the level of IMA. These results support the role of oxidative stress in AA.

It was challenging to find patients with AA who were suitable for inclusion, as it is not a common disease. In addition, these chemicals were expensive and difficult to obtain. In future studies, a larger number of patients could be evaluated, and it would be highly desirable to evaluate the cofactors of enzymes. Background pathophysiological data, lifestyles, and lifestyle-related diseases suffered by the studied subjects could be investigated in greater depth. Another limitation of our study was that patients were only evaluated at baseline and not after treatment with antioxidants. In addition, measuring and comparing total oxidant and total antioxidant capacity with IMA and the others was more reliable.

In conclusion, our study investigated the role of oxidative stress in the pathogenesis of AA via evaluation of IMA and other markers of the oxidant/antioxidant system, such as SOD, CAT, GSH-ST, and MDA, and contributing clinical risk factors. Our study found that the body was trying to increase the activity of antioxidant enzymes to protect itself against oxidative stress with GSH-ST. However, direct oxidative damage, changes in gene expression and dysfunction of some enzyme activity can be effective on the pathogenesis of AA. The amounts of oxidant products may not decrease due to the inability of SOD and CAT, despite an increase in enzyme activity of GSH-ST with high levels of ROS. The body may try to keep these products at normal levels to protect against harmful effects. However, this control can be very difficult in some situations, such as severe disseminated disease or the low level of enzyme activity in males. In particular, it would be useful to consider antioxidant support for protection against oxidant products of AA in males with long-lasting, disseminated severe disease. Follow-up on oxidative stress biomarkers and enzymatic action in AA may be useful and valuable, both before and during antioxidant treatment. To the best of our knowledge, this is one of the only studies to evaluate enzyme activity and biomarkers of oxidative stress in AA. Unlike other studies, we evaluated IMA in AA first. IMA can be detected in the condition of oxidative stress in this disease, and it has high potential as a biomarker when compared to other studied biomarkers. However, the usefulness of markers should be suggested by a pre/poststudy, intervention study, and/or outcome study. The results of our study cannot be generalized; therefore, additional studies are needed with larger groups of patients with AA.
